# An analytical approach for quantifying the influence of nanoparticle polydispersity on cellular delivered dose

**DOI:** 10.1098/rsif.2018.0364

**Published:** 2018-07-25

**Authors:** Stuart T. Johnston, Matthew Faria, Edmund J. Crampin

**Affiliations:** 1 Systems Biology Laboratory, School of Mathematics and Statistics, and Department of Biomedical Engineering, University of Melbourne, Parkville, Victoria 3010, Australia; 2 ARC Centre of Excellence in Convergent Bio-Nano Science and Technology, Melbourne School of Engineering, University of Melbourne, Parkville, Victoria 3010, Australia; 3 School of Medicine, Faculty of Medicine Dentistry and Health Sciences, University of Melbourne, Parkville, Victoria 3010, Australia

**Keywords:** delivered dose, sedimentation, diffusion, cellular association, nanoparticles

## Abstract

Nanoparticles provide a promising approach for the targeted delivery of therapeutic, diagnostic and imaging agents in the body. However, it is not yet fully understood how the physico-chemical properties of the nanoparticles influence cellular association and uptake. Cellular association experiments are routinely performed in an effort to determine how nanoparticle properties impact the rate of nanoparticle–cell association. To compare experiments in a meaningful manner, the association data must be normalized by the amount of nanoparticles that arrive at the cells, a measure referred to as the delivered dose. The delivered dose is calculated from a model of nanoparticle transport through fluid. A standard assumption is that all nanoparticles within the population are monodisperse, namely the nanoparticles have the same physico-chemical properties. We present a semi-analytic solution to a modified model of nanoparticle transport that allows for the nanoparticle population to be polydisperse. This solution allows us to efficiently analyse the influence of polydispersity on the delivered dose. Combining characterization data obtained from a range of commonly used nanoparticles and our model, we find that the delivered dose changes by more than a factor of 2 if realistic amounts of polydispersity are considered.

## 1. Introduction

Nanoparticles are a promising tool in the field of biomedicine, providing novel approaches for delivering agents in a targeted manner for therapeutic, imaging and diagnostic purposes [1–7]. For example, nano-sized polymer or mesoporous capsules can be synthesized and loaded with a therapeutic agent [5,7,8]. Ideally, the physico-chemical properties of the capsule allow for preferential interaction with a specific type of cell [5,7]. Alternatively, magnetic nanoparticles that accumulate in certain tissues can be used to detect and image disease [2,9]. However, significant questions remain regarding how altering the physico-chemical properties of nanoparticles during synthesis influences the cellular internalization of the nanoparticle, and hence influences the efficacy of the putative therapy [10].

Previous investigations have highlighted that nanoparticle properties such as size, shape, surface chemistry and surface charge all affect the interaction between cells and nanoparticles [10–17]. Furthermore, these properties have a more immediate effect. If the nanoparticles are immersed in a fluid that contains proteins, a layer of proteins will adsorb to the nanoparticle and form what is known as a protein corona [18–20]. This is particularly relevant for a variety of clinical applications, where nanoparticles are often immersed in whole blood [18,20]. The formation of the protein corona can significantly alter the size, density and surface charge of the nanoparticles [18,20,21]. Additionally, the protein corona can induce aggregation in a previously stable nanoparticle population, again significantly changing the size and density of the nanoparticles [19,22]. Hence the physico-chemical properties of the nanoparticles, which are typically characterized after the synthesis of the nanoparticles, change significantly after immersion in a protein-rich fluid. As the size and density of the nanoparticles are the key parameters that govern nanoparticle transport through fluid, it is critical that the nanoparticles are characterized post immersion. Accounting for the transport of nanoparticles through fluid allows for the delivered dose to be estimated; this is a measure of the number or mass of nanoparticles that arrive at the fluid–cell interface. The delivered dose provides a metric for comparing *in vitro* experiments for nanoparticles with different physico-chemical properties by normalizing against the number or mass of nanoparticles that interact with the cells [23–25].

While recent studies have begun to recognize the importance of accurately determining the delivered dose [19,23–30], one aspect that influences the transport of nanoparticles is typically neglected: the polydispersity of the nanoparticle population. Here we refer to polydispersity as variability in the physico-chemical properties of the nanoparticles within a population. In particular, we focus on the variability in the diameter of nanoparticles, which can arise from the synthesis process, the formation of aggregates or the formation of a protein corona, and the subsequent variability in nanoparticle density. The distribution of nanoparticle sizes present in a population of nanoparticles is routinely calculated, for instance, using dynamic light scattering (DLS) data [28,31,32]. However, these data are typically used only to measure the mean nanoparticle diameter and to ensure that the size distribution is unimodal. Nanoparticle transport through fluid is typically assumed to be governed by a combination of diffusion and sedimentation [27,33,34]. Both the diffusion and sedimentation parameters are nonlinear functions of diameter [24,34] and hence considering only the mean nanoparticle diameter may produce different estimates of the delivered dose, compared with considering the distribution of nanoparticle diameters. This effect may be compounded by the formation of a protein corona, the width of which depends on the diameter of the core nanoparticle [20,32,35]. Furthermore, the width of the protein layer has been demonstrated to follow a distribution rather than have a consistent size [35]. It is therefore critical for any delivered dose model to be able to account for variation in the core nanoparticle diameter, the nanoparticle density and the width of the protein corona. Previous mathematical and computational models of sedimentation and diffusion allow for the calculation of the delivered dose [28,34,36] and can be extended to analyse the polydisperse delivered dose through repeated numerical model realizations, performed with different parameters. Other nanoparticle transport models, which include random walk models [37], are more suited to examining polydispersity but are not amenable to analysis and require sufficient model realizations such that stochastic fluctuations do not dominate the solution behaviour. An analytic solution that can incorporate polydispersity and avoids the computational expense of repeatedly numerically solving the model equations is therefore beneficial. Furthermore, analytic solutions are amenable to analysis and provide insight into the influence of the physico-chemical parameters on delivered dose. Mahnama *et al.* [30] present an analytic solution for the original sedimentation and diffusion model, and examine the influence of the relative rates of sedimentation and diffusion on the delivered dose. While insightful, this approach is limited to monodisperse populations and a single boundary condition.

Here we present a modified model of sedimentation and diffusion that originates from the probability distribution of the position of an individual nanoparticle. We systematically build up this model to incorporate the variability in the nanoparticle population, and present the semi-analytic solution of the model. Using this solution, we demonstrate the difference between the delivered dose for a monodisperse population and the delivered dose for a polydisperse population, using the same mean nanoparticle diameter. We find that the delivered dose varies significantly with the standard deviation of the size distribution. We similarly compare the delivered dose for monodisperse and polydisperse populations for nanoparticles with a protein corona and nanoparticles that form aggregates, and find that the polydispersity should be considered to obtain an accurate delivered dose. To aid with comparison, we present a metric highlighting the influence of nanoparticle polydispersity on delivered dose for a suite of nanoparticle parameters and types of polydispersity. For each type of polydispersity, we conduct a theoretical analysis of the influence of the amount of polydispersity. Additionally, for each type of polydispersity, we present an experimental case study highlighting the influence of polydispersity for commonly used nanoparticles by combining our model with nanoparticle characterization data.

## 2. Model

**Figure 1.**
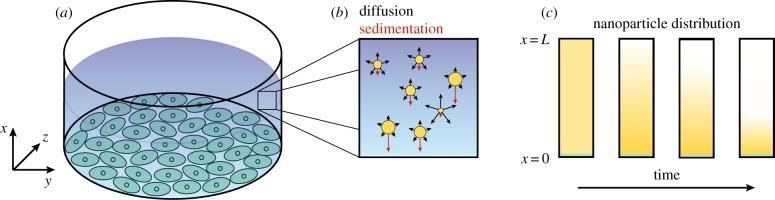
Schematic of the experimental geometry motivating the sedimentation and diffusion model. (*a*) Typical experimental geometry for an *in vitro* cellular association assay. (*b*) Processes governing nanoparticle transport in a fluid. Red arrows denote the direction of nanoparticle movement due to sedimentation and black arrows highlight the random direction of movement due to diffusion. The length of the arrows highlights the relative contributions of diffusion and sedimentation. (*c*) Evolution of the distribution of nanoparticles throughout the fluid over time. Orange denotes the presence of nanoparticles and green denotes the cell layer. (Online version in colour.)

Nanoparticle transport through a fluid is driven by a combination of sedimentation and diffusion [28,34,36]. Therefore, the evolution of the distribution for the position of an individual nanoparticle, *P*(*x*, *t*), is governed by
2.1
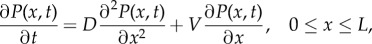
where *L* is the depth of the culture medium, *D* is the diffusivity of the nanoparticle and *V* is the sedimentation velocity of the nanoparticle. The approximate values of *D* and *V* arise from the Stokes–Einstein equation and Stokes' law, respectively [34]. Specifically, the diffusivity is given by
2.2
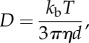
where *k*_b_ is the Boltzmann constant, *T* is the temperature of the fluid, *η* is the dynamic viscosity of the fluid and *d* is the diameter of the nanoparticle. The velocity is given by
2.3
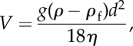
where *g* is the gravitational acceleration constant, *ρ* is the density of the nanoparticle and *ρ*_f_ is the density of the fluid. Equation (2.1) is valid for an experimental geometry where nanoparticles are initially approximately uniformly distributed in the plane parallel to the air–fluid interface, such as in the schematic presented in figure 1. The boundary conditions corresponding to this experimental geometry are
2.4
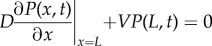
and
2.5
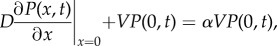
where *α* ≥ 0 represents the ability of the nanoparticles to associate with the cells. If *α* = 0 the nanoparticles do not associate with the cells and hence remain in the fluid, whereas as *α* increases the nanoparticles are increasingly able to associate with the cells and are therefore removed from the fluid. As equation (2.1) describes the position of an individual nanoparticle, the initial condition is a point source located at *x* = *x*_0_, that is,
2.6
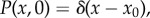
where *δ*(*x*) is the Dirac delta function. The model here is suitable for spherical nanoparticles. Non-spherical nanoparticles can be described through the use of dynamic shape factors, which account for the influence of shape on the diffusivity and sedimentation velocity [36]. While we do not consider non-spherical nanoparticles in this work, it is straightforward to adjust the diffusivity and sedimentation velocity to describe non-spherical nanoparticles.

We detail the full method of solution in the electronic supplementary material. We note that while the methodology is relatively standard [38] it is useful here as it does not rely on repeated numerical solutions of PDEs [30]. Briefly, we non-dimensionalize the model and solve the model using a combination of transform techniques and separation of variables [38]. We integrate the solution over both the initial particle locations and the domain, and subtract this from the initial amount of nanoparticles to obtain the delivered dose. To account for the nanoparticle polydispersity, we take a weighted integral of the delivered dose across the size distribution, where the weighting is given by the relative amounts of nanoparticles with a particular diameter. Ultimately, we calculate the delivered dose as a function of time and the size distribution parameters, typically given by the mean, *μ*, and the standard deviation, *σ*. We note that this approach is different from the approach considered by Rodriguez-Lorenzo *et al.* [37] to examine the influence of polydispersity. Our approach does not rely on the direct simulation of a large number of nanoparticles to obtain the average dosage, as we consider a purely deterministic approach. Hence this approach is not computationally intensive and can be efficiently employed to analyse the influence of polydispersity across a suite of nanoparticle characteristics. Additionally, our model has a flexible boundary condition that can describe instantaneous uptake of all nanoparticles at the cell boundary, zero uptake of nanoparticles at the cell boundary, or a specified uptake rate. The model of Rodriguez-Lorenzo *et al.* [37] can describe size-dependent adherence at the boundary; however, it is unclear how this would be implemented as a specific uptake rate.

## 3. Results

**Figure 2.**
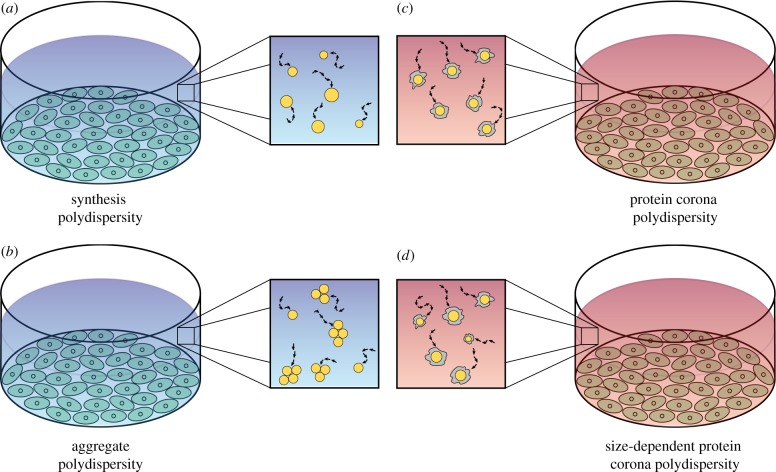
(*a*–*d*) Schematic of the four types of nanoparticle polydispersity considered, in the context of an *in vitro* cellular association experiment. Blue backgrounds denote protein-free media, whereas red backgrounds denote protein-rich media. Orange circles represent nanoparticles and grey shapes represent the protein corona. (Online version in colour.)

We use the solution derived in the previous section to examine the influence of four types of polydispersity, which we refer to as:

— *Synthesis polydispersity* (figure 2*a*), where the variation in nanoparticle diameters is due to the synthesis process. This is appropriate for a non-aggregating nanoparticle population in a fluid that does not contain proteins or if the nanoparticles are non-fouling, that is, proteins do not adsorb to the nanoparticles. We illustrate the influence of this polydispersity using data obtained from three types of polymer nanoparticles.
— *Aggregate polydispersity* (figure 2*b*), where nanoparticles are immersed in a fluid that does not contain proteins, and form aggregates consisting of different numbers of nanoparticles. Here we make the simplifying assumption that all nanoparticles are synthesized with the same diameter. We examine the impact of aggregate polydispersity by considering gold nanoparticle aggregation data.
— *Protein corona polydispersity* (figure 2*c*), where nanoparticles are immersed in a protein-rich fluid and a protein corona forms on the surface of the nanoparticles. Here the protein corona introduces the difference in hydrodynamic diameter, and we assume that all nanoparticles are synthesized with the same diameter. We examine the effect of protein corona polydispersity through the use of hydrodynamic diameter data obtained from polystyrene and silica nanoparticles after immersion in either a buffer solution or blood plasma.
— *Size-dependent protein corona polydispersity* (figure 2*d*), where nanoparticles are immersed in a protein-rich fluid and a protein corona forms on the surface of the nanoparticles. Here the nanoparticles are polydisperse after synthesis and the protein corona introduces further polydispersity. We use data obtained from a silica nanoparticle population to illustrate how this polydispersity can influence the delivered dose.

### 3.1. Synthesis polydispersity

**Figure 3.**
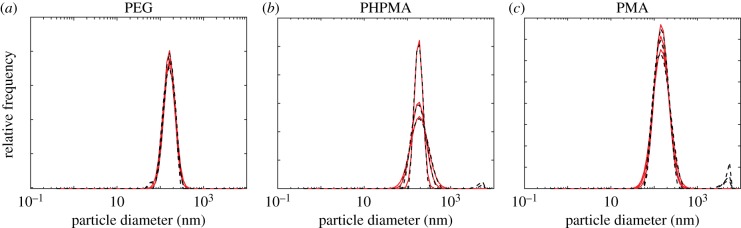
Comparison of size distributions obtained from DLS (black, dashed) and lognormal distributions using the mean and standard deviation values presented in table 1 (red) for (*a*) PEG nanoparticles, (*b*) PHPMA nanoparticles and (*c*) PMA nanoparticles. (Online version in colour.)

We first consider the synthesis polydispersity for three types of polymer nanoparticles created on mesoporous silica templates, after which the silica template is removed. For full experimental details, see Song *et al.* [39]. We note that, while these data are not new, they have not been interpreted in this manner previously. The size distributions obtained from DLS for poly(ethylene glycol) (PEG), poly(*N*-(2 hydroxypropyl)methacrylamide) (PHPMA) and poly(methacrylic acid) (PMA) nanoparticles are presented in figure 3 for three experimental replicates. As the size distribution is approximately normally distributed on a logarithmic scale, we fit a lognormal distribution to the data to obtain a value for the mean and standard deviation for the nanoparticle diameter, using Matlab's lsqnonlin function. The resulting lognormal distributions are superimposed on the DLS data in figure 3 and we observe that the DLS data are well described by a lognormal distribution. We present the mean and standard deviation obtained for the three replicates of the three types of nanoparticles in table 1. We note that for ease of interpretation we report the mean corresponding to the nanoparticle diameter rather than the mean of the lognormal distribution, *μ*_log_, and that 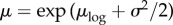. The standard deviation is left in dimensionless units such that the polydispersity between different nanoparticle populations can be compared easily. We note that we could use the polydispersity index (PDI) instead of the standard deviation, and that PDI = *σ*^2^_pop_/*μ*^2^, where *σ*_pop_ is the dimensional standard deviation and 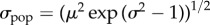.

**Table 1.** Mean and standard deviation values obtained from fitting a lognormal distribution to the DLS data presented in figure 3.

	replicate 1		replicate 2		replicate 3		average	
	mean (nm)	s.d.	mean (nm)	s.d.	mean (nm)	s.d.	mean (nm)	s.d.
PEG	181.61	0.29	186.53	0.33	191.50	0.31	186.55	0.31
PHPMA	196.04	0.23	261.86	0.47	225.35	0.38	227.75	0.36
PMA	183.48	0.40	196.15	0.44	180.45	0.37	186.69	0.41

**Figure 4.**
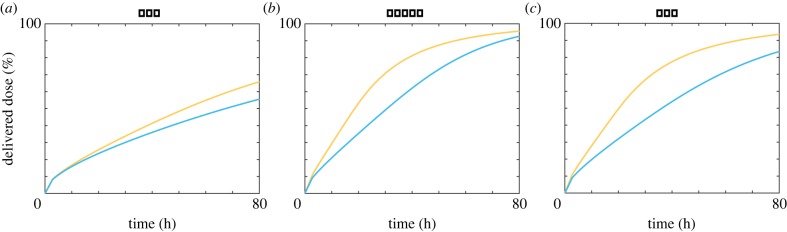
Time course of the mass delivered dose as a percentage of the administered dose for a monodisperse population (cyan) and a polydisperse population (orange) of (*a*) PEG nanoparticles, (*b*) PHPMA nanoparticles, (*c*) PMA nanoparticles. Parameters used are (*a*) *μ* = 186.55 nm, *σ* = 0.31, *ρ* = 1.13 g cm^−3^, (*b*) *μ* = 227.75 nm, *σ* = 0.36, *ρ* = 1.29 g cm^−3^, (*c*) *μ* = 186.69 nm, *σ* = 0.41, *ρ* = 1.33 g cm^−3^. (Online version in colour.)

We observe that for all three types of nanoparticle there is a significant range of nanoparticle diameters present in the DLS data. To quantify the influence of this polydispersity, we calculate the mass delivered dose for all three types of nanoparticles for a monodisperse population and a polydisperse population. Both populations have the same mean nanoparticle diameter, but the polydisperse population is described by a lognormal distribution with the standard deviation as defined in table 1. We present the time course of the mass delivered dose in figure 4. For each type of nanoparticle, we see that the polydisperse population has a higher delivered dose than the monodisperse population. This result is intuitive as a linear increase in nanoparticle diameter leads to a quadratic increase in sedimentation velocity. As such, the larger nanoparticles in the polydisperse population should arrive at the fluid–cell interface first. Smaller nanoparticles are more susceptible to random diffusive motion, and hence take longer to arrive at the fluid–cell interface. Additionally, the larger nanoparticles provide a proportionally greater contribution to the mass delivered dose, as mass scales cubically with diameter.

**Figure 5.**
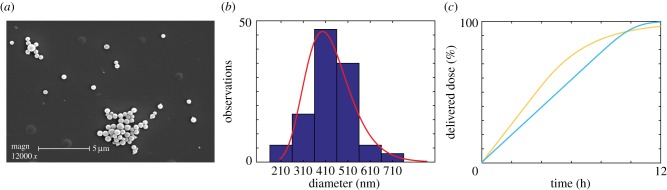
(*a*) SEM image of PEG @ mesoporous silica nanoparticles. (*b*) Histogram of measured nanoparticle diameters with the best-fit lognormal distribution superimposed. (*c*) Mass delivered dose for a monodisperse population (cyan) and a polydisperse population (orange) of PEG @ mesoporous silica nanoparticles obtained numerically. Parameters used are *μ* = 432.44 nm, *σ* = 0.24, *ρ* = 1.60 g cm^−3^. (Online version in colour.)

We perform a similar analysis using size distribution data obtained from scanning electron microscopy (SEM) images of PEG @ mesoporous silica nanoparticles, and present the results in figure 5. Experimental details can be found in the electronic supplementary material. We observe similar polydispersity present in the SEM measurements of PEG nanoparticles, as in the DLS data of polymer nanoparticles considered previously. Again, we find that the mass delivered dose is enhanced in the presence of size polydispersity.

To quantify the influence of the degree of polydispersity, we introduce the normalized half-dose time (NHDT), which is the time taken for half of the administered dose to be delivered for the polydisperse population, compared with the time taken for half of the administered dose to be delivered for the monodisperse population. The NHDT, denoted *H*(*μ*, *σ*), can be calculated according to
3.1
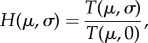
where *T*(*μ*, *σ*) is the time taken for half of the administered dose to be delivered for a nanoparticle population with size distribution parameters *μ* and *σ*. If the NHDT has a value greater than 1 this implies that the polydispersity inhibits the delivered dose, whereas if the NHDT has a value less than 1 this implies that the polydispersity enhances the delivered dose. For all results in this work, the parameters *T* = 310 K, *ρ*_f_ = 1.00 g cm^−3^, *ρ*_pr_ = 1.33 g cm^−3^, *α* = 10^8^, *η* = 6.9 × 10^−4^ kg/(m · s), *n*_max_ = 50 and Δ_*θ*_ = 30 are used to calculate the time course of the delivered dose. For a detailed explanation of the parameters, see the electronic supplementary material.

**Figure 6.**
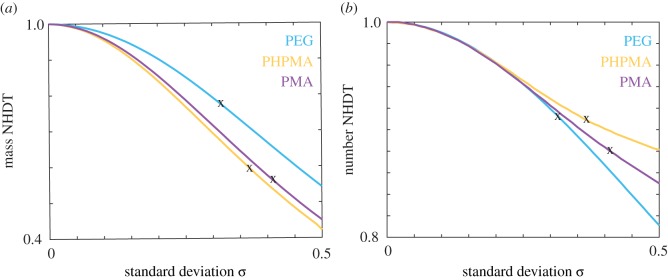
(*a*) Mass NHDT, (*b*) number NHDT for the PEG (cyan), PHPMA (orange) and PMA (purple) nanoparticles for a range of standard deviation values. Crosses correspond to experimental data. Parameters used are *t*_end_ = 80 h, *μ*_PEG_ = 186.55 nm, *ρ*_PEG_ = 1.13 g cm^−3^, *μ*_PHPMA_ = 227.75 nm, *ρ*_PHPMA_ = 1.29 g cm^−3^, *μ*_PMA_ = 186.69 nm, *ρ*_PMA_ = 1.33 g cm^−3^. (Online version in colour.)

In figure 6, we examine how the NHDT changes with the standard deviation for PEG, PHPMA and PMA nanoparticles with the respective mean nanoparticle diameters presented in table 1. Here we present the NHDT for both the mass delivered dose and the number delivered dose. For all nanoparticle types and both measures of delivered dose, we observe that the NHDT decreases as the standard deviation increases, and that *H*(*μ*, 0.5) < 0.6 for all three types of nanoparticles for the mass delivered dose. As the PDI increases as the standard deviation increases, an increase in PDI would also correspond to a decrease in the NHDT. If we consider the standard deviation values obtained from the DLS data, denoted by black crosses in figure 6*a*, we observe a reduction in the time taken for half of the mass to be delivered by 20–50%. This reduction highlights the importance of accounting for the polydispersity, as the standard assumption of monodispersity can result in dosage estimates that are significantly different from the actual delivered dose.

**Figure 7.**
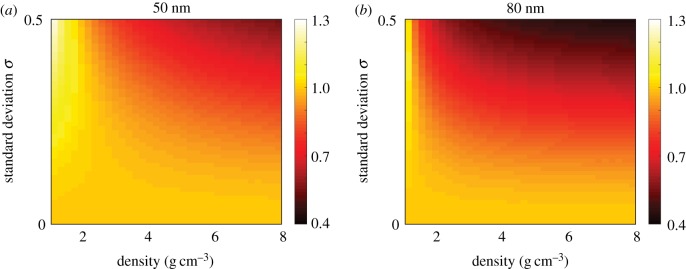
Mass NHDT for a suite of nanoparticle densities and standard deviation values for nanoparticles with a mean diameter of (*a*) 50 nm, (*b*) 80 nm. Parameters used are *t*_end_ = 120 h. (Online version in colour.)

To generalize, we extend our analysis by examining the mass NHDT for nanoparticles with a suite of density, mean diameter and standard deviation values, and present the results in figure 7. Note that the density and size parameters used are representative of commonly used nanoparticles but are not explicitly obtained from experimental data. For both 50 and 80 nm nanoparticles and for all density values, we observe that the NHDT decreases with the standard deviation. This decrease is more pronounced for higher density nanoparticles. It is therefore critical to incorporate the polydispersity in a population of nanoparticles to calculate the delivered dose, as it is possible that conclusions drawn from cellular association data may be incorrect if comparisons are made between experiments where the amount of polydispersity differs between experiments.

### 3.2. Aggregate polydispersity

We next consider how aggregate polydispersity can influence the delivered dose. An increase in the number of nanoparticles per aggregate changes the effective diameter of the aggregate and, additionally, changes the density depending on the fractal dimension, *D*_f_, of the aggregate. A higher *D*_f_ value represents nanoparticles that form an aggregate that is less porous, and hence has less medium within the internal structure of the aggregate. The aggregate diameter can be expressed as
3.2
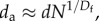
where *N* is the distribution of the number of nanoparticles per aggregate. Note that this neglects the packing factor as presented by Sterling, as this is typically less important than the fractal dimension [11]. The density of the aggregate, *ρ*_a_, is
3.3
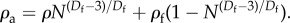
As *D*_f_ → 3, *ρ*_a_ approaches the original nanoparticle density as the amount of medium within the internal structure of the aggregate decreases. Note that both *d*_a_ and *ρ*_a_ are affected by the aggregate polydispersity as *N* follows a distribution.

**Figure 8.**
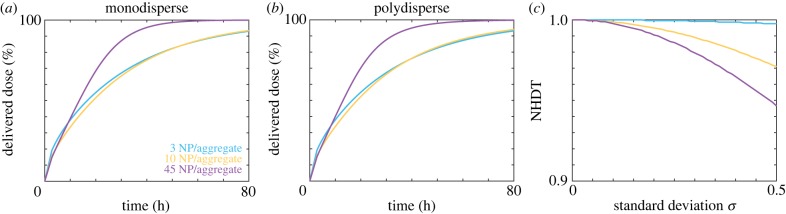
(*a*) Delivered dose for a monodisperse population of 16 nm Au nanoparticles with 3 (cyan), 10 (orange) and 45 (purple) nanoparticles per aggregate. (*b*) Delivered dose for a polydisperse population of 16 nm Au nanoparticles with an average of 3 (cyan), 10 (orange) and 45 (purple) nanoparticles per aggregate. (*c*) NHDT for a polydisperse population of 16 nm Au nanoparticles with an average of 3 (cyan), 10 (orange) and 45 (purple) nanoparticles per aggregate for a range of standard deviation values. Parameters used are (*a*–*c*) *ρ* = 19.32 g cm^−3^, *t*_end_ = 80 h, (*b*) *σ* = 0.5. (Online version in colour.)

To investigate aggregate polydispersity, we consider 16 nm gold (Au) nanoparticles as presented by Albanese & Chan [11], and present the time course of the delivered dose in figure 8*a*,*b* for a monodisperse population and a polydisperse population, respectively, with a mean of 3, 10 and 45 nanoparticles per aggregate. We obtain the mean number of nanoparticles per aggregate from the reported aggregate size and with the assumption of a fractal dimension of 2.1 [11,37]. Intuitively, we observe that an increase in the number of nanoparticles per aggregate corresponds to an increase in delivered dose, as the diameter of the aggregate increases and hence the rate of sedimentation increases. However, the delivered dose appears to be similar for the monodisperse and polydisperse populations. To analyse the difference between the monodisperse and polydisperse populations, we present the NHDT for a range of standard deviation values in figure 8*c*. For aggregate polydispersity, the mass and number NHDT are interchangeable as we make the assumption that the nanoparticles are synthesized with a consistent diameter. We observe that the NHDT decreases with both the standard deviation and number of nanoparticles per aggregate. However, in general, the decrease in the NHDT associated with aggregate polydispersity is significantly less pronounced than the decrease in the NHDT associated with synthesis polydispersity. Interestingly, this is in contrast with the results presented by Rodriguez-Lorenzo *et al.* [37], who found that aggregate polydispersity inhibits the number delivered dose.

**Figure 9.**
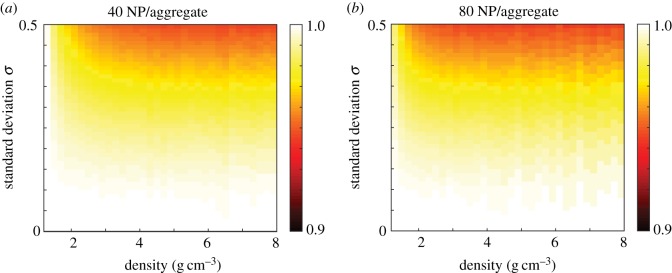
NHDT for aggregate polydisperse 30 nm nanoparticles with a mean number of nanoparticles per aggregate of (*a*) 40, (*b*) 80 for a range of standard deviation and density values. Parameters used are *d* = 30 nm, *t*_end_ = 120 h. (Online version in colour.)

We next calculate the NHDT for a suite of mean number of nanoparticles per aggregate, density and standard deviation values, and present the results in figure 9. Similar to the results obtained from the 16 nm Au nanoparticles, we observe that the aggregate polydispersity only influences the delivered dose slightly compared with the synthesis polydispersity for 30 nm nanoparticles. For a mean of 40 and 80 nanoparticles per aggregate the NHDT decreases by less than 10% due to polydispersity. This result is intuitive, if we consider the influence of aggregation on the sedimentation velocity, which increases with both diameter and density. While the diameter of the aggregate is larger than the individual nanoparticle, the density decreases as the aggregate has a porous structure and hence the impact of the increase in diameter on the sedimentation velocity is reduced. Taking into account the diameter-dependent reduction in density, the sedimentation velocity scales according to *d*_a_^*D*_f_−1^ for aggregate polydispersity compared with *d*^2^ for synthesis polydispersity. As the fractal dimension is typically around 2 [11,37], this suggests that the sedimentation velocity scales approximately linearly with diameter. Hence the assumption of monodispersity does not significantly influence the delivered dose, as the velocity of a nanoparticle with diameter equal to the mean diameter value is similar to the mean of the velocities obtained from the distribution of nanoparticle diameters due to the approximately linear relationship between the sedimentation velocity and nanoparticle diameter. This suggests that the variability in the number of nanoparticles per aggregate is less important than the variability in nanoparticle diameter, though it should still be considered if the aim is to compare experiments in a consistent and meaningful manner.

### 3.3. Protein corona polydispersity

We next consider the influence of protein corona polydispersity, where the width of the protein corona follows a distribution and the nanoparticle core has a fixed diameter. The hydrodynamic diameter of the nanoparticle is therefore
3.4
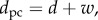
where *w* is the width of the protein corona. We make the assumption that *w* follows a lognormal distribution and that *w* does not change throughout the experiment. It has been reported previously that the protein corona forms within minutes [20], which is significantly faster than the hours typically required for the nanoparticles to sediment and diffuse to the cell layer. Hence it is reasonable to assume that the protein corona has a constant width throughout the transport process. The density of the proteins composing the protein corona layer, *ρ*_pr_, affects the overall density of the nanoparticle, which can be calculated as
3.5
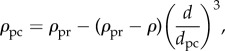
where *ρ*_pc_ is the effective density of the nanoparticle and adsorbed protein corona. Here the mass and number delivered dose are interchangeable as the mass delivered dose is determined by the core nanoparticle, which we assume to have constant width for this type of polydispersity.

**Figure 10.**
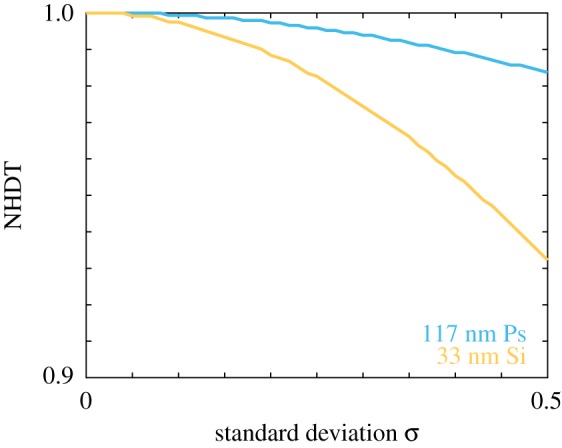
NHDT for a protein corona polydisperse population of 117 nm Ps nanoparticles (cyan) and 33 nm Si nanoparticles (orange), with a mean protein corona width of 39 nm and 42 nm, respectively, for a range of standard deviation values. Parameters used are *t*_end_ = 80 h, *ρ*_Ps_ = 1.05 g cm^−3^, *ρ*_Si_ = 2.33 g cm^−3^. (Online version in colour.)

Characterization of nanoparticles in fluid with and without proteins suggests that the width of the adsorbed protein corona can be greater than the original diameter of the nanoparticle [20]. Here we consider polystyrene (Ps) nanoparticles and silica (Si) nanoparticles, as reported by Tenzer *et al.* [20], where 117 nm Ps and 33 nm Si nanoparticles underwent an increase in hydrodynamic diameter of 39 nm and 42 nm, respectively. Again, we examine the influence of polydispersity by calculating the NHDT for a range of standard deviation values for both the Ps and Si nanoparticles, where a protein corona forms on the surface of the nanoparticles and the width of the protein corona follows a lognormal distribution. We present the NHDT for a range of standard deviation values in figure 10. Similar to the change in NHDT due to aggregate polydispersity, we observe that the reduction in NHDT due to protein corona polydispersity is less than 10% for the range of standard deviation values considered for both nanoparticles. While the formation of the protein corona can significantly influence the delivered dose, the results in figure 10 suggest that the variability in the protein corona width does not significantly change the delivered dose. Although the NHDT does not change with protein corona polydispersity as significantly as size polydispersity, it is still important to account for the putative change in the calculated delivered dose to ensure that experimental comparisons are valid.

**Figure 11.**
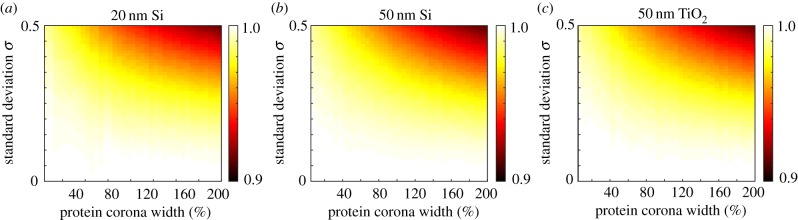
NHDT for a protein corona polydisperse (*a*) 20 nm Si, (*b*) 50 nm Si, (*c*) 50 nm TiO_2_ nanoparticle population for a range of standard deviation and density values. Parameters used are *t*_end_ = 80 h, *ρ*_Si_ = 2.33 g cm^−3^, *ρ*_TiO_2__ = 4.23 g cm^−3^. (Online version in colour.)

To determine whether the presence of polydispersity can have a more pronounced impact on the delivered dose for other types of nanoparticles, we calculate the NHDT for a suite of standard deviation and protein corona width values, and we present the corresponding results in figure 11. The parameters considered correspond to 20 nm and 50 nm Si nanoparticles and 50 nm titanium dioxide (TiO_2_) nanoparticles. Note that we report the protein corona width as a percentage of the core nanoparticle diameter. We observe that in all cases the presence of a polydisperse protein corona results in an NHDT that decreases with polydispersity. Interestingly, the impact on the delivered dose is similar for both 50 nm nanoparticles, which suggests that the density is not a significant factor for protein corona polydispersity. However, we again observe that the NHDT is reduced by at most 10% due to protein corona polydispersity. Similar to aggregate polydispersity, the limited amount of reduction can be attributed to the decrease in density that accompanies the increase in the hydrodynamic diameter. The sedimentation velocity increases proportional to *d*^2^_pc_, whereas the effective density contains a term that is proportional to *d*^−3^_pc_. It is important to note that the *d*^−3^_pc_ term does not dominate the value of the effective density as it is bounded by the density of the nanoparticle core and protein corona, and hence the sedimentation velocity does not scale inversely with *d*_pc_.

### 3.4. Size-dependent protein corona polydispersity

Finally, we consider size-dependent protein corona polydispersity. The purpose of this is twofold. First, to examine how the influence of polydispersity is compounded by considering two processes that introduce polydispersity, namely the synthesis polydispersity and the protein corona polydispersity. Second, to highlight the importance of characterizing the nanoparticles after immersion in a protein-filled fluid. If the nanoparticles are characterized in a dry state, then the information about the size of the nanoparticle after formation of the protein corona is lost, which can have a significant impact on the delivered dose, and hence lead to conclusions about nanoparticle association or uptake that could be attributed to the change in the nanoparticle properties. The diameter and density of these nanoparticles is again described by equations (3.4) and (3.5), albeit now both *d* and *w* are described by a size distribution. We make the simplifying assumption that the mean protein corona width is a fixed proportion of the mean nanoparticle diameter. For example, a 50 nm nanoparticle and an 80 nm nanoparticle from the same population would have a mean protein corona width of 25 nm and 40 nm, respectively, if the mean protein corona width is half of the core nanoparticle diameter. Further, we assume that both *d* and *w* have the same standard deviation.

**Figure 12.**
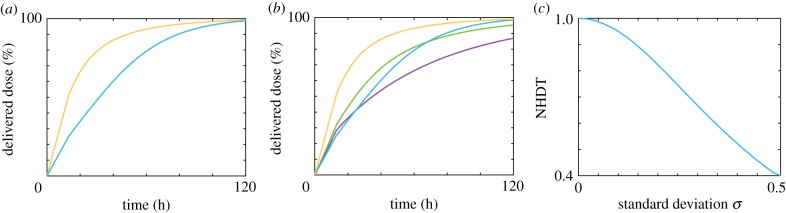
(*a*) Mass delivered dose for a size-dependent protein corona polydisperse (orange) and monodisperse (cyan) Si nanoparticle population with a mean diameter of 65 nm and a mean protein corona width of 130 nm. (*b*) Mass delivered dose for a size-dependent protein corona polydisperse (orange) and monodisperse (cyan) Si nanoparticle population with a synthesis polydisperse (green) and monodisperse (purple) population with a mean diameter of 65 nm. (*c*) Comparison of the mass NHDT for a size-dependent protein corona population of Si nanoparticles with a mean diameter of 65 nm and a mean protein corona width of 130 nm for a range of standard deviation values. Parameters used are *t*_end_ = 80 h, *ρ*_Si_ = 2.33 g cm^−3^; (*a*,*b*) *σ* = 0.5. (Online version in colour.)

To examine the influence of size-dependent protein corona polydispersity, we consider the mesoporous silica nanoparticles reported by Feiner-Gracia *et al.* [35]. The authors provide measurements of both the core nanoparticle diameter and the width of the adsorbed protein corona [35]. The protein corona width is approximately double the core nanoparticle diameter [35]. Similarly, the reported standard deviation, in absolute terms, for the protein corona width is approximately double the standard deviation for the core nanoparticle diameter. Hence the assumption that *d* and *w* have the same standard deviation is valid here as the reported standard deviations are the same proportion of the corresponding nanoparticle diameter. We calculate the time course of the mass delivered dose for a monodisperse and polydisperse population of nanoparticles, as well as the NHDT for a range of standard deviation values, and present the results in figure 12. In figure 12*a* we observe, similar to the synthesis polydispersity, that there is a clear difference in the time course of the mass delivered dose for the monodisperse and polydisperse populations. This difference is particularly pronounced at short time, which arises from the contributions of the nanoparticles with an above-average diameter and sedimentation velocity to the mass delivered dose. Such nanoparticles arrive at the fluid–cell interface earlier due to the increase in sedimentation velocity, and have an enhanced contribution to the mass delivered dose due to the additional volume of the nanoparticles, compared with nanoparticles in the monodisperse population. We highlight the importance of characterizing the nanoparticles in a hydrated state by presenting the time course of the mass delivered dose for the monodisperse and polydisperse population with and without the protein corona in figure 12*b*. We observe that the delivered dose is significantly lower if the delivered dose is calculated with the nanoparticle properties obtained from characterizing the nanoparticles in a non-hydrated state. The decrease in the NHDT with the standard deviation, presented in figure 12*c*, reinforces the observation that size-dependent protein corona polydispersity significantly impacts the delivered dose. Interestingly, the addition of a protein corona of varying width to the core nanoparticles with varying diameter does not compound the influence of polydispersity. This result is perhaps unsurprising as the results presented in figures 10 and 11 demonstrate that the protein corona polydispersity has minimal influence on the delivered dose, and hence we expect the change in the delivered dose to be associated with the polydispersity of the core nanoparticles rather than the protein corona.

**Figure 13.**
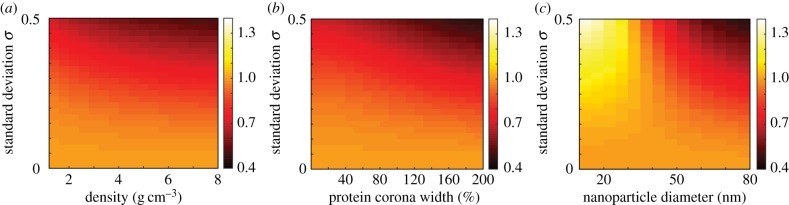
Mass NHDT for a size-dependent protein corona polydisperse population for a range of standard deviation and (*a*) density values, (*b*) protein corona width values, (*c*) core nanoparticle diameter values. Parameters used are *t*_end_ = 120 h, (*a*) *d* = 50 nm, *w* = 50 nm, (*b*) *d* = 50 nm, *ρ*_TiO2_ = 4.32 g cm^−3^ and (*c*) *w* = *d*, *ρ*_Si_ = 2.33 g cm^−3^. (Online version in colour.)

Finally, we extend our investigation of size-dependent protein corona polydispersity to examine how the NHDT changes with standard deviation for a suite of nanoparticle density, protein corona width and mean diameter values. The NHDT values calculated for a range of values for each nanoparticle characteristic are presented in figure 13. We observe that the NHDT changes significantly with the standard deviation for each nanoparticle characteristic. For example, in figure 13*a*, for nanoparticles with a density of 8 g cm^−3^, we see a 60% reduction in the NHDT due to size-dependent protein corona polydispersity, whereas for nanoparticles with a density of 2 g cm^−3^ and the same diameter and protein corona width we see a 40% reduction in the NHDT. This implies that dense nanoparticles are more strongly influenced by the presence of polydispersity. Similarly, in figure 13*b*, we observe a 60% reduction in the NHDT for a protein corona width equal to 200% of the core nanoparticle diameter, compared with a 40% reduction for a protein corona with a width of 50% of the core nanoparticle diameter. If we vary the core nanoparticle diameter for silica nanoparticles where the protein corona width is equal to the core nanoparticle diameter and calculate the NHDT, as presented in figure 13*c*, we observe that for 30 nm and smaller nanoparticles the delivered dose is reduced due to the polydispersity. This is because nanoparticle transport for small and light nanoparticles is dominated by diffusion as both the diameter and the difference between the density of the nanoparticle and the fluid is small. Hence an increase in diameter due to polydispersity will increase the rate of sedimentation but this rate is still small compared with the rate of diffusion. The inhibition of delivered dose for sufficiently small nanoparticles due to polydispersity is in contrast with the change in delivered dose for nanoparticles with a diameter greater than 30 nm, where the polydispersity enhances the delivered dose. As it is possible for the delivered dose to be either significantly enhanced or inhibited in the presence of polydispersity, it is critical to accurately evaluate the delivered dose by accounting for the polydispersity within the nanoparticle population, otherwise conclusions drawn from experimental comparisons may be invalid.

## 4. Discussion and conclusion

We have presented a modified model of the sedimentation and diffusion of nanoparticles that can account for the polydispersity of the nanoparticle population. Furthermore, we derive an analytic solution for this model with a flexible boundary condition at the fluid–cell interface that allows for the direct calculation of the delivered dose. As our solution technique is analytic, it allows us to analyse the influence of polydispersity on the delivered dose in a methodical and efficient manner. Previous approaches have required multiple realizations of the underlying numerical model, and it is therefore computationally demanding to explore the impact of polydispersity on the delivered dose across a suite of nanoparticle parameters [36,37]. By contrast, the time course of the delivered dose for a polydisperse population of nanoparticles can be obtained from our solution in less than a second on a desktop computer. We consider four types of polydispersity that can arise in cellular association experiments. However, the various forms of polydispersity are typically neglected with respect to their impact on cellular delivered dose, which may affect the validity of comparisons between experiments performed under different conditions or with different nanoparticles. To quantify the impact of the polydispersity, we introduce a metric describing the time taken for half of the administered dose to arrive at the fluid–cell interface for a polydisperse population, compared with the time taken for a monodisperse population: the NHDT. Our approach and new metric allow us to quantify how different aspects of variability in the physico-chemical parameters of nanoparticles can manifest themselves in the variability of the cellular association data.

We find that all four types of polydispersity influence the delivered dose and hence should be considered when evaluating the delivered dose. In particular, considering solely the polydispersity arising from the nanoparticle synthesis can account for a 60% reduction in the NHDT. If the cellular association is proportional to the delivered dose [27], then the polydisperse population would show a significantly higher association with the cells. This highlights how critical it is to account for the polydispersity in a nanoparticle population. For example, if cellular association data are compared across two experiments and one population shows more cellular association, and polydispersity is not considered, it is unclear whether the additional cellular association is due to the polydispersity or to a stronger affinity that the nanoparticles may have with the cell population. Polydispersity arising due to either variability in aggregate size or protein corona width, under the assumption of a monodisperse core nanoparticle population, can account for a 10% reduction in the NHDT, for commonly used nanoparticles. While these types of polydispersity do not influence the delivered dose as significantly as the synthesis polydispersity, they should still be considered when calculating the delivered dose. Finally, we find that if both the nanoparticle core and protein corona vary in diameter and width, respectively, as observed in the recent work by Feiner-Gracia *et al.* [35], the impact of polydispersity is further compounded, though this is not significant compared with the impact of synthesis polydispersity. By contrast, the difference between the synthesis polydispersity delivered dose and the size-dependent protein corona polydispersity delivered dose highlights the importance of characterizing nanoparticles in a hydrated state, as the change in hydrodynamic diameter due to the formation of the protein corona significantly impacts the rates of sedimentation and diffusion, and subsequently impacts the delivered dose. A summary of the impact of the different types of polydispersity is presented in table 2, as well as the relationship between the impact of the polydispersity and the different nanoparticle characteristics. The results presented in table 2 highlight the importance of considering the polydispersity to robustly and accurately calculate the delivered dose, particularly for high-density nanoparticles where the polydispersity is due to the synthesis process.

**Table 2.** Summary of the impact of each type of polydispersity for different types of nanoparticles (NP), and the relationship between impact and nanoparticle size, number of nanoparticles per aggregate and protein corona width. A single tick denotes a nanoparticle for which polydispersity has a slight (0–10%) impact on delivered dose, two ticks denotes a nanoparticle for which polydispersity has a significant (10–40%) impact on delivered dose and three ticks denotes a nanoparticle for which polydispersity has a dramatic (more than 40%) impact on delivered dose. Plus symbols (+) denote an increase in the impact of polydispersity with the corresponding parameter, whereas tilde symbols (∼) denote no change in the impact. There is no decrease in the impact of polydispersity with a parameter. ‘n.a.’ refers to a parameter that is not relevant for a particular type of polydispersity. Polymer nanoparticles refer to low-density nanoparticles (less than 3 g cm^−3^) and inorganic nanoparticles refer to high-density nanoparticles (more than 3 g cm^−3^).

	impact		size		NP/aggregate		protein corona width	
polydispersity type	polymer	inorganic	polymer	inorganic	polymer	inorganic	polymer	inorganic
synthesis	✓✓	✓✓✓	+	+	n.a.	n.a.	n.a.	n.a.
aggregate	✓	✓	∼	∼	∼	∼	n.a.	n.a.
protein corona	✓	✓	∼	∼	n.a.	n.a.	+	+
size-dependent protein corona	✓✓	✓✓✓	+	+	n.a.	n.a.	+	+

The work presented here has implications for the experimental protocol required to reliably and robustly compare cellular association data, as well as for previously published experimental data where polydispersity has not been considered. One benefit to our approach is that it is relatively straightforward to retroactively calibrate a size distribution to existing experimental data to examine whether the conclusions made are influenced by the polydispersity within the nanoparticle population. Hence it would be instructive to investigate potential relationships between nanoparticle properties and cellular association, once the polydisperse delivered dose is accounted for. Previous investigations typically either neglect the delivered dose, do not characterize the nanoparticles in a hydrated state or do not consider the influence of the polydispersity on the delivered dose. As we have demonstrated, it is critical to accurately determine the delivered dose using both the properties of the hydrated nanoparticles and the polydispersity to be able to robustly compare experiments and draw meaningful conclusions.

This works highlights several avenues for future research. We have considered a relatively simple boundary condition for the fluid–cell interface, which implies that the cell receptors do not become saturated with nanoparticles. While this is a relatively common assumption, there exists an opportunity to examine the influence of more sophisticated boundary conditions, such as a boundary condition where the cell receptors have a maximum nanoparticle concentration or a boundary condition that incorporates internalization kinetics [36]. Alternatively, it could be instructive to investigate whether cellular association data could provide insight into the degree of polydispersity in the nanoparticle population. As it is not necessarily straightforward to characterize the nanoparticle population in a hydrated state, particularly if the nanoparticles tend to form aggregates, it would be of interest to determine whether the size distribution of the nanoparticles can be estimated reliably from cellular association data.

## Supplementary Material

Supplementary Information

## Data accessibility

The experimental images, image analysis algorithm and parameters can be found in the electronic supplementary material.
