# Supplementary material for: An analytical approach for quantifying the influence of nanoparticle polydispersity on cellular delivered dose
Source: J R Soc Interface. 2018 Jul 25;15(144):20180364. doi: 10.1098/rsif.2018.0364 (PMC6073649; doi:10.1098/rsif.2018.0364)
Supplement: Supplementary Information [file rsif20180364supp1.pdf]

An analytical approach for quantifying the influence of nanoparticle polydispersity on cellular delivered dose
Supplementary Information

Stuart T. Johnston[1,2], Matthew Faria [1,2] and Edmund J. Crampin[1,2,3]

1. Systems Biology Laboratory, School of Mathematics and Statistics, and Department of Biomedical Engineering, University of Melbourne, Parkville, Victoria 3010, Australia.
2. ARC Centre of Excellence in Convergent Bio-Nano Science and Technology, Melbourne School of Engineering, University of Melbourne, Parkville, Victoria 3010, Australia.
3. School of Medicine, Faculty of Medicine Dentistry and Health Sciences, University of Melbourne, Parkville, Victoria 3010, Australia.

Model Solution

The evolution of the distribution for the position of an individual, $P(x,t)$, is governed by

$$\frac{\partial P(x,t)}{\partial t} = D\frac{\partial^2 P(x,t)}{\partial x^2} + V\frac{\partial P(x,t)}{\partial x}, \tag{1}$$

where D is the diffusivity of the particle and V is the velocity of the particle. The values of these parameters arise from the Stokes-Einstein equation and Stokes' law, respectively. Specifically, the diffusivity is given by

$$D = \frac{k_b T}{3\pi\eta d}, \tag{2}$$

where k_b is the Boltzmann constant, T is the temperature of the fluid, η is the dynamic viscosity of the fluid and d is the diameter of the particle. The velocity is given by

$$V = \frac{g(\rho-\rho_f)d^2}{18\eta}, \tag{3}$$

where g is the gravitational acceleration constant, ρ is the density of the particle and ρ_f is the density of the fluid. The boundary conditions corresponding to the experimental geometry of interest are

$$D\left.\frac{\partial P(x,t)}{\partial x}\right|_{x=L} + VP(L,t) = 0, \tag{4}$$

and

$$D\left.\frac{\partial P(x,t)}{\partial x}\right|_{x=0} + VP(0,t) = \alpha VP(0,t), \tag{5}$$

where $\alpha \geq 0$ represents the ability of the nanoparticles to associate with the cells. For a single nanoparticle, we assume a point source initial condition at $x = x_0$,

$$P(x,0) = \delta(x-x_0). \tag{6}$$

To solve Equation (1) subject to Equations (4)-(6) we first non-dimensionalise the system by introducing the variables

$$\chi = \frac{x}{L}, \qquad \text{Pe} = \frac{VL}{D}, \qquad \text{and } \tau = \frac{tV}{L}, \tag{7}$$

where Pe is the Peclet number. The model therefore becomes

$$\frac{P(\chi,\tau)}{\partial \tau} = \frac{1}{\text{Pe}}\frac{\partial^2 P(\chi,\tau)}{\partial \chi^2} + \frac{\partial P(\chi,\tau)}{\partial \chi}, \tag{8}$$

subject to the initial condition

$$P(\chi,0) = \delta\left(\chi - \frac{x_0}{L}\right), \tag{9}$$

and the boundary conditions

$$\left.\frac{\partial P(\chi,\tau)}{\partial \chi}\right|_{\chi=1} + \mathrm{Pe}P(1,\tau) = 0, \tag{10}$$

and

$$\left.\frac{\partial P(\chi,\tau)}{\partial \chi}\right|_{\chi=0} + \mathrm{Pe}(1-\alpha)P(0,\tau) = 0. \tag{11}$$

We next introduce the new variable

$$Q(\chi,\tau) = P(\chi,\tau)\exp\left(\frac{\mathrm{Pe}}{4}\tau + \frac{\mathrm{Pe}}{2}\chi\right). \tag{12}$$

Equations (8)-(11) can be expressed in terms of this new variable, which gives rise to the governing equation

$$\frac{\partial Q(\chi,\tau)}{\partial \tau} = \frac{1}{\mathrm{Pe}}\frac{\partial^2 Q(\chi,\tau)}{\partial \chi^2}, \tag{13}$$

with initial condition

$$Q(\chi,0) = \exp\left(\frac{\mathrm{Pe}}{2}\chi\right)\delta\left(\chi - \frac{x_0}{L}\right), \tag{14}$$

and the boundary conditions

$$\left.\frac{\partial Q(\chi,\tau)}{\partial \chi}\right|_{\chi=1} + \frac{\mathrm{Pe}}{2}Q(1,\tau) = 0, \tag{15}$$

and

$$\left.\frac{\partial Q(\chi,\tau)}{\partial \chi}\right|_{\chi=0} + \frac{\mathrm{Pe}}{2}(1-2\alpha)Q(0,\tau) = 0. \tag{16}$$

To solve this, we follow a standard separation of variables approach and assume that functions $X(\chi)$ and $T(\tau)$ exist such that

$$Q(\chi,\tau) = X(\chi)T(\tau). \tag{17}$$

Equation (13) therefore implies that

$$\frac{X''(\chi)}{X(\chi)} = \mathrm{Pe}\frac{T'(\tau)}{T(\tau)} = -\lambda^2, \tag{18}$$

where λ^2 are the eigenvalues of the system. Note that the dash refers to an ordinary derivative. The solution for $T(\tau)$ is straightforward and is

$$T(\tau) = \exp\left(\frac{-\lambda^2}{\mathrm{Pe}}\tau\right). \tag{19}$$

The solution for $X(\chi)$ depends on the sign of the eigenvalues. Interestingly, depending on the value of α, the number of non-positive eigenvalues change. Assuming $\lambda^2 = 0$,

$$X(\chi) = A(\chi) + B. \tag{20}$$

Upon substitution into the boundary conditions, it can be seen that there are no non-trivial solutions that satisfy the boundary conditions unless

$$\mathrm{Pe}(1-2\alpha) - 4\alpha = 0, \tag{21}$$

in which case there is a single non-trivial solution and hence the leading eigenvalue is zero. If we instead assume $\lambda^2 < 0$,

$$X(\chi) = C_1\cosh(\lambda\chi) + C_2\sinh(\lambda\chi). \tag{22}$$

Again, there are no non-trivial solutions that satisfy the boundary conditions unless

$$\mathrm{Pe}(1-2\alpha) - 4\alpha > 0, \tag{23}$$

in which case there is one non-trivial solution, which gives rise to a negative leading eigenvalue that can be obtained from the solution to

$$(\mathrm{Pe}^2(1-2\alpha)-4\lambda^2)\sinh(\lambda)-4\lambda\mathrm{Pe}\alpha\cosh(\lambda)=0. \tag{24}$$

Finally, if $\lambda^2>0$,

$$X(\chi)=D_1\cos(\lambda\chi)+D_2\sin(\lambda\chi). \tag{25}$$

There are an infinite number of eigenvalues that satisfy the boundary conditions for this form of the solution, and the eigenvalues can be obtained from the solutions to

$$(\mathrm{Pe}^2(1-2\alpha)+4\lambda^2)\sin(\lambda)-4\lambda\mathrm{Pe}\alpha\cos(\lambda)=0. \tag{26}$$

There is exactly one root of Equation (26), λ_n, on each interval $[n\pi,(n+1)\pi]$, except for the case where

$$\mathrm{Pe}(1-2\alpha)-4\alpha\geq 0, \tag{27}$$

where the leading eigenvalue is non-positive as discussed previously, and hence (26) has no solution on $[0,\pi]$.

Hence there are three solution regimes for the model depending on the values of α and Pe:
If $\mathrm{Pe}(1-2\alpha)-4\alpha<0$,

$$Q(\chi,\tau)=\sum_{n=0}^{\infty}A_n\left[\sin(\lambda_n\chi)-\frac{2\lambda_n}{\mathrm{Pe}(1-2\alpha)}\cos(\lambda_n\chi)\right]\exp\left(\frac{-\lambda_n^2}{\mathrm{Pe}}\tau\right). \tag{28}$$

If $\mathrm{Pe}(1-2\alpha)-4\alpha=0$,

$$Q(\chi,\tau)=A_0(1-2\alpha\chi)+\sum_{n=1}^{\infty}A_n\left[\sin(\lambda_n\chi)-\frac{2\lambda_n}{\mathrm{Pe}(1-2\alpha)}\cos(\lambda_n\chi)\right]\exp\left(\frac{-\lambda_n^2}{\mathrm{Pe}}\tau\right). \tag{29}$$

If $\mathrm{Pe}(1-2\alpha)-4\alpha=0$,

$$\begin{aligned}Q(\chi,\tau)=&A_0\left[\sinh(\lambda_0\chi)-\frac{2\lambda_0}{\mathrm{Pe}(1-2\alpha)}\cosh(\lambda_0\chi)\right]\exp\left(\frac{\lambda_0^2}{\mathrm{Pe}}\tau\right)\\&+\sum_{n=1}^{\infty}A_n\left[\sin(\lambda_n\chi)-\frac{2\lambda_n}{\mathrm{Pe}(1-2\alpha)}\cos(\lambda_n\chi)\right]\exp\left(\frac{-\lambda_n^2}{\mathrm{Pe}}\tau\right).\end{aligned} \tag{30}$$

To obtain the values for the coefficients A_n we use generalised Fourier Series and the initial condition. That is,

$$\begin{aligned}A_n&=\frac{\int_0^1 Q(\chi,0)\left[\sin(\lambda_n\chi)-\frac{2\lambda_n}{\mathrm{Pe}(1-2\alpha)}\cos(\lambda_n\chi)\right]\,d\chi}{\int_0^1\left[\sin(\lambda_n\chi)-\frac{2\lambda_n}{\mathrm{Pe}(1-2\alpha)}\cos(\lambda_n\chi)\right]^2\,d\chi}\\&=\frac{4\lambda_n\mathrm{Pe}^2(1-2\alpha)^2\exp\left(\frac{\mathrm{Pe}x_0}{2L}\right)\left[\sin\left(\lambda_n\frac{x_0}{L}\right)-\frac{2\lambda_n}{\mathrm{Pe}(1-2\alpha)}\cos\left(\lambda_n\frac{x_0}{L}\right)\right]}{\mathrm{Pe}^2(1-2\alpha)^2(2\lambda_n-\sin(2\lambda_n))+4\lambda_n\mathrm{Pe}(1-2\alpha)(\cos(2\lambda_n)-1)+4\lambda_n(2\lambda_n^2+\lambda_n\sin(2\lambda_n))}.\end{aligned} \tag{31}$$

For the case where $\mathrm{Pe}(1-2\alpha)-4\alpha=0$,

$$\begin{aligned}A_0&=\frac{\int_0^1 Q(\chi,0)\left[1-2\alpha\chi\right]\,d\chi}{\int_0^1\left[1-2\alpha\chi\right]^2\,d\chi}\\&=\frac{\exp\left(\frac{\mathrm{Pe}x_0}{2L}\right)\left(1-\frac{2\alpha x_0}{L}\right)}{1-2\alpha+\frac{4\alpha^2}{3}}.\end{aligned} \tag{32}$$

For the case where $\mathrm{Pe}(1-2\alpha)-4\alpha>0$,

$$\begin{aligned}A_0&=\frac{\int_0^1 Q(\chi,0)\left[\sinh(\lambda_0\chi)-\frac{2\lambda_0}{\mathrm{Pe}(1-2\alpha)}\cosh(\lambda_0\chi)\right]\,d\chi}{\int_0^1\left[\sinh(\lambda_0\chi)-\frac{2\lambda_0}{\mathrm{Pe}(1-2\alpha)}\cosh(\lambda_0\chi)\right]^2\,d\chi}\\&=\frac{4\lambda_0\mathrm{Pe}^2(1-2\alpha)^2\exp\left(\frac{\mathrm{Pe}x_0}{2L}\right)\left[\sinh\left(\lambda_0\frac{x_0}{L}\right)-\frac{2\lambda_0}{\mathrm{Pe}(1-2\alpha)}\cosh\left(\lambda_0\frac{x_0}{L}\right)\right]}{\mathrm{Pe}^2(1-2\alpha)^2(\sinh(2\lambda_0)-2\lambda_0)+4\lambda_0\mathrm{Pe}(1-2\alpha)(1-\cosh(2\lambda_0))+4\lambda_0(2\lambda_0^2+\lambda_0\sinh(2\lambda_0))}.\end{aligned} \tag{33}$$

Transforming $Q(\chi,\tau)$ back to $P(x,t)$ we obtain

$$P(x,t)=\sum_{n=0}^{\infty}A_n\left[\sin\left(\lambda_n\frac{x}{L}\right)-\frac{2\lambda_n}{\mathrm{Pe}(1-2\alpha)}\cos\left(\lambda_n\frac{x}{L}\right)\right]\exp\left(-\left(\frac{\lambda_n^2D}{L^2}+\frac{V^2}{4D}\right)t-\frac{V}{2D}x\right), \tag{34}$$

for $\mathrm{Pe}(1-2\alpha)-4\alpha<0$, where $A_n, n\geq 0$ is defined in Equation (31). For $\mathrm{Pe}(1-2\alpha)-4\alpha=0$,

$$P(x,t)=\left\{A_0(1-2\alpha\frac{x}{L})+\sum_{n=1}^{\infty}A_n\left[\sin\left(\lambda_n\frac{x}{L}\right)-\frac{2\lambda_n}{\mathrm{Pe}(1-2\alpha)}\cos\left(\lambda_n\frac{x}{L}\right)\right]\exp\left(\frac{-\lambda_n^2D}{L^2}t\right)\right\}\exp\left(\frac{-V^2}{4D}t-\frac{V}{2D}x\right), \tag{35}$$

where $A_n,\ n\geq 1$ is defined in Equation (31) and A_0 is defined in Equation (32). For $\mathrm{Pe}(1-2\alpha)-4\alpha>0$,

$$\begin{aligned}P(x,t)=&\left\{A_n\left[\sinh\left(\lambda_0\frac{x}{L}\right)-\frac{2\lambda_0}{\mathrm{Pe}(1-2\alpha)}\cosh\left(\lambda_0\frac{x}{L}\right)\right]\exp\left(\frac{\lambda_0^2D}{L^2}t\right)\right.\\&\left.+\sum_{n=1}^{\infty}A_n\left[\sin\left(\lambda_n\frac{x}{L}\right)-\frac{2\lambda_n}{\mathrm{Pe}(1-2\alpha)}\cos\left(\lambda_n\frac{x}{L}\right)\right]\exp\left(\frac{-\lambda_n^2D}{L^2}t\right)\right\}\exp\left(\frac{-V^2}{4D}t-\frac{V}{2D}x\right),\end{aligned} \tag{36}$$

where $A_n,\ n\geq 1$ is defined in Equation (31) and A_0 is defined in Equation (33).

$P(x,t)$ describes the probability distribution of the position of a particle initially located at $x=x_0$ and we note that there is an implicit dependence on x_0. However, we are typically interested in more than a single particle. Hence we consider that there is initially some distribution of particles with the same properties throughout the fluid. In the absence of additional information we make the assumption that the particles are distributed uniformly at random. The nanoparticle distribution throughout the fluid, $\hat{P}(x,t)$, is therefore given by

$$\hat{P}(x,t)=\frac{1}{L}\int_0^L P(x,t)\ dx_0. \tag{37}$$

Furthermore, we are interested in calculating the delivered dose, $U(t)$, which is the proportion of the initially administered dose that has arrived at the cell population. This can be calculated by evaluating the difference between the initial nanoparticle mass present in the fluid and the nanoparticle mass present in the fluid at time t. Specifically,

$$U(t)=\int_0^L \hat{P}(x,0)\ dx-\int_0^L \hat{P}(x,t)\ dx. \tag{38}$$

We can calculate these integrals analytically. For $\mathrm{Pe}(1-2\alpha)-4\alpha<0$,

$$\begin{aligned}\int_0^L \hat{P}(x,t)\ dx=&\sum_{n=0}^{\infty}B_n\exp\left(-\left(\frac{\lambda_n^2D}{L^2}+\frac{V^2}{4D}\right)t\right)\times\Bigg[\\&\left\{\frac{\exp\left(\frac{\mathrm{Pe}}{2}\right)\left[\left(\frac{\mathrm{Pe}}{2L}-\frac{2\lambda_n^2}{\mathrm{Pe}(1-2\alpha)L}\right)\sin(\lambda_n)-\left(\frac{\lambda_n}{(1-2\alpha)L}+\frac{\lambda_n}{L}\right)\cos(\lambda_n)\right]+\frac{\lambda_n}{L}+\frac{\lambda_n}{(1-2\alpha)L}}{\frac{\lambda_n^2}{L^2}+\frac{\mathrm{Pe}^2}{4L^2}}\right\}\times\\&\left\{\frac{\exp\left(\frac{-\mathrm{Pe}}{2}\right)\left[\left(\frac{\lambda_n}{(1-2\alpha)L}-\frac{\lambda_n}{L}\right)\cos(\lambda_n)-\left(\frac{\mathrm{Pe}}{2L}+\frac{2\lambda_n^2}{\mathrm{Pe}(1-2\alpha)L}\right)\sin(\lambda_n)\right]+\frac{\lambda_n}{L}-\frac{\lambda_n}{(1-2\alpha)L}}{\frac{\lambda_n^2}{L^2}+\frac{\mathrm{Pe}^2}{4L^2}}\right\}\Bigg],\end{aligned} \tag{39}$$

where, for $n\geq 0$,

$$B_n=\frac{4\lambda_n\mathrm{Pe}^2(1-2\alpha)^2}{\mathrm{Pe}^2(1-2\alpha)^2(2\lambda_n-\sin(2\lambda_n))+4\lambda_n\mathrm{Pe}(1-2\alpha)(\cos(2\lambda_n)-1)+4\lambda_n(2\lambda_n^2+\lambda_n\sin(2\lambda_n))}. \tag{40}$$

For $\mathrm{Pe}(1-2\alpha)-4\alpha=0$,

$$\int_0^L \hat{P}(x,t)\;dx = \exp\left(\frac{-V^2}{4D}t\right) \times \Bigg\{ B_0\left[\frac{16L^4\left\{\exp\left(\frac{\mathrm{Pe}}{2}\right)\left[\frac{\mathrm{Pe}}{2L}+\frac{2\alpha}{V}-\frac{\mathrm{Pe}\alpha}{V}\right]-\frac{\mathrm{Pe}}{2L}-\frac{2\alpha}{L}\right\}\left\{\exp\left(\frac{-\mathrm{Pe}}{2}\right)\left[\frac{\mathrm{Pe}\alpha}{L}-\frac{\mathrm{Pe}}{2L}+\frac{2\alpha}{L}\right]+\frac{\mathrm{Pe}}{2L}-\frac{2\alpha}{L}\right\}}{\mathrm{Pe}^4}\right]$$
$$+\sum_{n=1}^{\infty} B_n \exp\left(\frac{-\lambda_n^2 D}{L^2}t\right)\times\Bigg[$$
$$\left\{\frac{\exp\left(\frac{\mathrm{Pe}}{2}\right)\left[\left(\frac{\mathrm{Pe}}{2L}-\frac{2\lambda_n^2}{\mathrm{Pe}(1-2\alpha)L}\right)\sin(\lambda_n)-\left(\frac{\lambda_n}{(1-2\alpha)L}+\frac{\lambda_n}{L}\right)\cos(\lambda_n)\right]+\frac{\lambda_n}{L}+\frac{\lambda_n}{(1-2\alpha)L}}{\frac{\lambda_n^2}{L^2}+\frac{\mathrm{Pe}^2}{4L^2}}\right\}\times$$
$$\left\{\frac{\exp\left(\frac{-\mathrm{Pe}}{2}\right)\left[\left(\frac{\lambda_n}{(1-2\alpha)L}-\frac{\lambda_n}{L}\right)\cos(\lambda_n)-\left(\frac{\mathrm{Pe}}{2L}+\frac{2\lambda_n^2}{\mathrm{Pe}(1-2\alpha)L}\right)\sin(\lambda_n)\right]+\frac{\lambda_n}{L}-\frac{\lambda_n}{(1-2\alpha)L}}{\frac{\lambda_n^2}{L^2}+\frac{\mathrm{Pe}^2}{4L^2}}\right\}\Bigg]\Bigg\}, \tag{41}$$

where, for $n \geq 1$, B_n is defined in Equation (40) and

$$B_0 = \frac{1}{1-2\alpha+\frac{4\alpha^2}{3}}. \tag{42}$$

For $\mathrm{Pe}(1-2\alpha)-4\alpha>0$,

$$\int_0^L \hat{P}(x,t)\;dx = \exp\left(\frac{-V^2}{4D}t\right) \times \Bigg\{ B_0 \exp\left(\frac{\lambda_0^2 D}{L^2}t\right)\times\Bigg[$$
$$\left\{\frac{\exp\left(\frac{\mathrm{Pe}}{2}\right)\left[\left(\frac{\mathrm{Pe}}{2L}+\frac{2\lambda_0^2}{\mathrm{Pe}(1-2\alpha)L}\right)\sinh(\lambda_0)-\left(\frac{\lambda_0}{(1-2\alpha)L}+\frac{\lambda_0}{L}\right)\cosh(\lambda_0)\right]+\frac{\lambda_0}{L}+\frac{\lambda_0}{(1-2\alpha)L}}{\frac{\mathrm{Pe}^2}{4L^2}-\frac{\lambda_0^2}{L^2}}\right\}\times$$
$$\left\{\frac{\exp\left(\frac{-\mathrm{Pe}}{2}\right)\left[\left(\frac{2\lambda_0^2}{\mathrm{Pe}(1-2\alpha)L-\frac{\mathrm{Pe}}{2L}}\right)\sinh(\lambda_0)+\left(\frac{\lambda_0}{(1-2\alpha)L}-\frac{\lambda_0}{L}\right)\cosh(\lambda_0)\right]+\frac{\lambda_0}{L}-\frac{\lambda_0}{(1-2\alpha)L}}{\frac{\mathrm{Pe}^2}{4L^2}-\frac{\lambda_0^2}{L^2}}\right\}\Bigg]$$
$$+\sum_{n=1}^{\infty} B_n \exp\left(-\left(\frac{\lambda_n^2 D}{L^2}+\frac{V^2}{4D}\right)t\right)\times\Bigg[$$
$$\left\{\frac{\exp\left(\frac{\mathrm{Pe}}{2}\right)\left[\left(\frac{\mathrm{Pe}}{2L}-\frac{2\lambda_n^2}{\mathrm{Pe}(1-2\alpha)L}\right)\sin(\lambda_n)-\left(\frac{\lambda_n}{(1-2\alpha)L}+\frac{\lambda_n}{L}\right)\cos(\lambda_n)\right]+\frac{\lambda_n}{L}+\frac{\lambda_n}{(1-2\alpha)L}}{\frac{\lambda_n^2}{L^2}+\frac{\mathrm{Pe}^2}{4L^2}}\right\}\times$$
$$\left\{\frac{\exp\left(\frac{-\mathrm{Pe}}{2}\right)\left[\left(\frac{\lambda_n}{(1-2\alpha)L}-\frac{\lambda_n}{L}\right)\cos(\lambda_n)-\left(\frac{\mathrm{Pe}}{2L}+\frac{2\lambda_n^2}{\mathrm{Pe}(1-2\alpha)L}\right)\sin(\lambda_n)\right]+\frac{\lambda_n}{L}-\frac{\lambda_n}{(1-2\alpha)L}}{\frac{\lambda_n^2}{L^2}+\frac{\mathrm{Pe}^2}{4L^2}}\right\}\Bigg]\Bigg\}, \tag{43}$$

where, for $n \geq 1$, B_n is defined in Equation (40) and

$$B_0 = \frac{4\lambda_0 \mathrm{Pe}^2(1-2\alpha)^2}{\mathrm{Pe}^2(1-2\alpha)^2(\sinh(2\lambda_0)-2\lambda_0)+4\lambda_0\mathrm{Pe}(1-2\alpha)(1-\cosh(2\lambda_0))+4\lambda_0(2\lambda_0^2+\lambda_0\sinh(2\lambda_0))}. \tag{44}$$

Equation (38) describes the delivered dose for a nanoparticle population with a single set of physicochemical characteristics, that is, the same density and diameter. We are interested in the delivered dose across a polydisperse population of nanoparticles. If the nanoparticle diameters follow a size distribution $S(\eta|\underline{\theta})$ where $\underline{\theta}$ are the shape parameters that govern the distribution then the delivered dose for this population, $U_p(t)$ is given by

$$U_p(t) = \int_\eta S(\eta|\underline{\theta})U(t)\ d\eta, \tag{45}$$

noting that the value of η influences V, D and Pe depending on the type of polydispersity considered. Note that this delivered dose corresponds to the number delivered dose.

For the mass delivered dose we add an additional weighting function that corresponds to the relative volume of each nanoparticle, and normalise by the total volume of the population,

$$U_{\text{mass}}(t) = \frac{\frac{4\pi}{24}\int_\eta d(\eta)^3 S(\eta|\underline{\theta})U(t)\ d\eta}{\frac{4\pi}{24}\int_\eta d(\eta)^3 S(\eta|\underline{\theta})\ d\eta}, \tag{46}$$

where $d(\eta)$ is the diameter of the nanoparticle.

Method of solution

As the integral in Equation (46) cannot be solved for an arbitrary size distribution, we use a numerical approximation of the integral consisting of Equation (38) evaluated at Δ_θ values of the size distribution. We truncate the infinite series in each solution after n_{max} terms and note that the solution is insensitive to the inclusion of additional terms. To obtain the eigenvalues defined by the solutions to Equations (24) and (26) we use Matlab's `fzero` function, with the interval of possible function roots defined as previously. We note that in specific cases, corresponding to nanoparticles where sedimentation is significantly larger than diffusion, oscillations in the solution can appear at short times, due to the presence of Gibbs phenomena. In these cases, a numerical solution should be considered.

Experimental details

To obtain the scanning electron microscopy data we incubated 400 nm PEG (40kDa) @ mesoporous silica nanoparticles in Milli-Q in a 12 well plate containing a silica wafer. Particles composed of PEG were synthesised using a mesoporous silica template according to a previous published method [1, 2], and the silica template was not removed from the nanoparticle. The nanoparticles were taken from a stock solution at 4.4×10^6 nanoparticles/μL, and 45 μL of this solution was added to 1455 μL of Milli-Q. Prior to incubation in the well, the nanoparticle/Milli-Q solution was vortexed and briefly sonicated to assure uniform distribution throughout the solution. Particles were undisturbed for 4 h and allowed to settle. After 4 h of incubation, fluid was removed from the well. The remaining silica wafer was then gold-sputtered and imaged using scanning electron microscopy (Philips XL30 FESEM) with a 15 kV beam. Images of the nanoparticles atop the silica wafer were captured at 12,000x magnification.

To obtain the size distribution of the nanoparticles we chose three representative SEM images, shown in Figures S1(a)-(c). The size of the nanoparticles was detected and measured automatically using Matlab's `imfindcircles` function with a minium radius of 7 pixels, a maximum radius of 30 pixels and a edge threshold detection value of 0.3. We overlay the detected circles in Figures S1(d)-(f). Red circles correspond to detected circles that are manually removed due to inaccuracy, while green circles correspond to detected circles that contribute to the measured size distribution (Main manuscript, Figure 5(b)). Finally, we fit a lognormal distribution to the histogram of measured nanoparticle diameters.

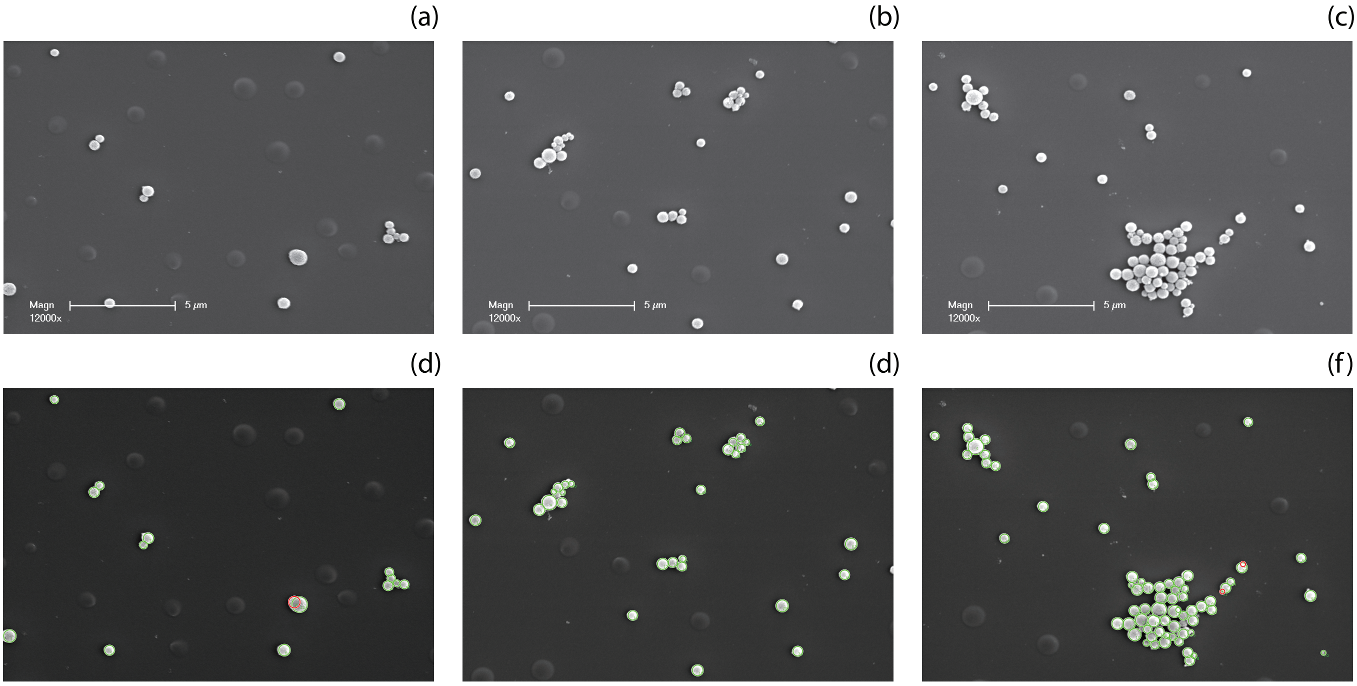

Figure 1: Scanning electron microscopy images of 400nm PEG @ mesoporous silica nanoparticles. Green circles represent nanoparticles detected and measured by our image analysis algorithm. Red circles correspond to detected nanoparticles that are manually removed, and hence not measured, as inspection revealed these to be false positives.

References

[1] Bjornmalm M, Cui J, Bertleff-Zieschang N, Song D, Faria M, Rahim MA, Caruso F. Nanoengineering particles through template assembly. Chemistry of Materials. 2016;29(1):289-306.

[2] Cui J, De Rose R, Alt K, Alcantara S, Paterson BM, Liang K, Hu M, Richardson JJ, Yan Y, Jeffery CM, Price RI. Engineering poly (ethylene glycol) particles for improved biodistribution. ACS Nano. 2015;9(2):1571-80.
